# Randomized Trial of Self-Selected Music Intervention on Pain and Anxiety in Emergency Department Patients with Musculoskeletal Back Pain

**DOI:** 10.5811/westjem.34871

**Published:** 2025-06-25

**Authors:** Charlotte E. Goldfine, Jenna M. Wilson, Jenson Kaithamattam, Mohammad Adrian Hasdianda, Kate Mancey, Alexander Rehding, Kristin L. Schreiber, Peter R. Chai, Scott G. Weiner

**Affiliations:** * Brigham and Women’s Hospital, Department of Emergency Medicine, Boston, Massachusetts; † Brigham and Women’s Hospital, Department of Anesthesiology, Boston, Massachusetts; ‡ Utrecht University, Department of Media and Culture Studies, Netherlands; § Harvard University, Department of Media and Culture Studies, Utrecht University, Netherlands; ¶ The Fenway Institute, Boston, Massachusetts; || Dana Farber Cancer Institute, Department of Psychosocial Oncology and Palliative Care, Boston, Massachusetts; # Massachusetts Institute of Technology, The Koch Institute for Integrated Cancer Research, Cambridge, Massachusetts

## Abstract

**Introduction:**

Acute musculoskeletal back pain is a frequent cause of emergency department (ED) visits, often with suboptimal relief from standard treatments. Recent evidence suggests listening to music may modulate pain and anxiety. In this pilot randomized controlled trial, we evaluated the impact of a brief session of patient-selected music vs noise cancellation on pain severity and anxiety in patients presenting to the ED with back pain.

**Methods:**

Patients with acute back pain completed a baseline survey to assess demographics, medication information, and psychosocial factors. The ED patients were randomized to listen to self-selected music or to noise cancellation (control). Patients rated their pain and anxiety (0–10) before and immediately after the intervention. We used analyses of covariance to examine whether post-intervention pain and anxiety differed between the groups, while controlling for baseline trait pain catastrophizing. A mediation analysis was conducted to explore the role of post-intervention anxiety as a mediator of the group difference in post-intervention pain.

**Results:**

Forty patients were enrolled with an average age of 47.2 years (range 21 – 81). and 27 patients (68%) were female. At baseline, patients in the music group reported higher pain catastrophizing compared to patients in the noise cancellation group. There were no other group differences in baseline characteristics. Post-intervention, patients in the music group reported significantly lower anxiety (3.0 ± 0.7 vs 5.5 ± 0.7, *P* = 0.016) and pain severity (6.1 ± 0.4 vs.7.5 ± 0.4, *P* = 0.037) compared to the noise cancellation group. A mediation analysis showed that post-intervention anxiety partially mediated the association between intervention group (music vs noise cancellation) and post-intervention pain.

**Conclusion:**

A brief session of self-selected music resulted in lower pain and anxiety scores than noise cancellation among patients with musculoskeletal back pain in the ED. Patients who listened to music reported lower post-intervention anxiety, which partially contributed to lower post-intervention pain severity.

## INTRODUCTION

Back pain affects approximately 540 million people globally and is the leading cause of years lived with disability worldwide.^1^ Acute exacerbations or new-onset low back pain can be distressing and result in temporary and long-term disability. While the etiology of back pain is variable, the most common reason individuals experience back pain is musculoskeletal.^2^ When pain persists despite conventional over-the-counter (OTC) pharmacotherapy, or when the reason for pain is uncertain, individuals may use the emergency department (ED) for evaluation. There are more than 2.6 million annual ED visits in the United States for back pain, and back pain accounts for up to 4.4% of all ED visits worldwide.^3^

Low back pain can be difficult to manage in the ED. The pillars of pharmacological treatment, nonsteroidal anti-inflammatory drugs (NSAID) and acetaminophen, have been compared to various other therapeutic options such as opioids, benzodiazepines, and musculoskeletal relaxants like methocarbamol, baclofen and cyclobenzaprine. 4,5 Even with recommended pharmacotherapy, individuals may experience suboptimal relief and request other analgesics, including opioids for pain. Finally, the experience of pain is modulated by psychosocial factors, such as anxiety; this may be particularly true in the ED, with anxiety more strongly contributing to perceptions of pain. 6–8 Therefore, there is a critical need to develop adjuvant therapies to address musculoskeletal back pain that could provide acute relief in the ED.

One potential strategy that demonstrates promise in mitigating the experience of pain is music.^9^ Music is unique in that it is nearly universally familiar and acceptable across different sociodemographic backgrounds, easily accessible, non-stigmatizing, and poses little to no risk of adverse effects.^10^ Prior studies have explored the use of music to mitigate anxiety surrounding painful procedures or to address postoperative pain and anxiety.^11^ Despite the potential for an effect, the use of music as an adjunct for treatment of acute pain in the ED has not been widely or systematically studied. In a prior study of patients who were admitted to an ED observation unit, we found that listening to 10 minutes of relaxing music was associated with decreased acute pain scores.^12^ In the same study, we also demonstrated that music decreased anxiety, which is a known psychological modulator associated with worse pain.^12^ This reduction in anxiety was correlated with reduced pain scores. In a laboratory setting with healthy adults, we additionally demonstrated that participants’ pain threshold and tolerance were significantly higher (ie, less pain sensitive) when listening to their self-selected favorite music compared to control conditions,^13^ suggesting that allowing patients to self-select their own music may be a promising strategy for reducing pain.

In the current study, we evaluated the effect of a brief session of patient-selected music vs noise cancellation on pain severity and anxiety in patients presenting to the ED with acute musculoskeletal back pain. We also explored user experiences and acceptability of using an adjunctive music intervention in the ED.

**Population Health Research Capsule**

What do we already know about this issue?

*Musculoskeletal back pain is a frequent reason for pain-related ED visits. Listening to music may help decrease pain and anxiety*.

What was the research question?

*Does patient-selected music impact pain severity and anxiety in patients presenting to the ED with back pain?*

What was the major finding of the study?

*Music resulted in lower pain (6.1 ± 0.4 vs.7.5 ± 0.4, P =.037) and anxiety (3.0 ± 0.7 vs. 5.5 ± 0.7, P =.016) scores*.

How does this improve population health?

*Listening to music in the ED may be an adjunctive tool in the armamentarium of non-pharmacologic interventions for musculoskeletal back pain*.

## METHODS

### Participants and Procedure

We recruited patients presenting to the emergency department (ED) with a chief complaint of acute back pain at Brigham and Women’s Hospital in Boston, MA, a tertiary care, urban, academic ED with approximately 65,000 annual visits. Sample size for this pilot study was chosen to be 40 based on prior studies.^12^,^14^ Research assistants identified and approached eligible patients to explain the study and gauge potential interest in participating. Inclusion criteria included reporting an initial pain severity rating of ≥5/10 at triage and being ≥18 years of age. Participants were excluded if they had hearing loss or were non-English speaking. Study procedures were approved by the Mass General Brigham Human Research Committee (protocol number 2022P000102). Formal written consent was obtained from all participants.

After providing informed consent, patients completed a baseline survey with basic demographic information, medication use, and questionnaires assessing psychosocial factors and clinical pain. Immediately prior to the music intervention, patients provided ratings for their current pain severity and level of anxiety, using a point scale from 0–10. Next, patients were randomized using REDCap (hosted at Mass General Brigham) in a 1:1 fashion to a music or noise cancellation (control) group. In the music group, patients were lent a pair of headphones and asked to select an artist or genre of music from a streaming music service (Spotify Premium) to listen to for 10 minutes. Once selected, participants were not able to skip songs or change their music selection. In the noise cancellation group, patients were lent a pair of noise cancellation headphones to wear for 10 minutes. Immediately after each intervention, all patients completed a follow-up assessment to reassess current pain and anxiety ratings and were then also asked questions about their experience with the interventions.

### Measures

Patients completed self-report measures assessing demographic characteristics (age, sex, race/ethnicity), self-reported use of opioids and non-opioid analgesics prior to presenting to the ED and completed validated brief questionnaires. We used the Patient Reported Outcome Measurement Information System (PROMIS) short forms to assess anxiety, depression, and sleep disturbance over the prior week.^15^ The Perceived Stress Scale (PSS) was used to measure subjective stress experienced over the past week.^16^ We measured trait pain catastrophizing, which involves negative pain-related cognitions, using the Pain Catastrophizing Scale (PCS).^17^

Prior to the intervention, patients reported their current pain at rest and during movement using a numeric rating scale (NRS) of 0–10 (0= no pain, 10= worst pain). These two pain ratings were averaged for a total pain severity index score. Patients also reported their current level of anxiety using a NRS (0=no anxiety, 10=worst anxiety). Immediately after the completion of the 10-minute interventions, all patients again reported their current pain severity and anxiety.

Analgesic medications administered during the ED visit, as well as upon discharge from the ED, were abstracted from patients’ electronic health records and converted to morphine milligram equivalents (MME) using a publicly available opioid conversion calculator. 18

We conducted a post-intervention survey to collect information about how patients normally used music or a music app for relaxation, how much they liked the intervention (1=strongly disliked, 5=strongly liked), whether the intervention changed the way they felt/thought about pain, and whether they thought they could deal with their pain using music therapy in combination with non-opioid medications.

### Data Analysis

Descriptive data is presented as means and standard deviations for continuous variables and as percentages for categorical variables. We used independent samples *t*-tests and chi-square analyses to explore whether patients randomized to the music group differed compared to those randomized to the noise cancellation group based on baseline characteristics. Patient characteristics that significantly differed (*P*<.05) between the two groups at baseline were included as covariates in subsequent analyses. We used analyses of covariance (ANCOVA) to examine whether post-intervention ratings of pain severity and anxiety significantly differed between the music and noise cancellation groups, while controlling for baseline covariates.

We conducted a follow-up exploratory analysis to explore whether post-intervention anxiety mediated, or contributed to, the group (music vs noise cancellation) difference in post-intervention pain severity. First, Pearson correlations were conducted to examine the association between post-intervention anxiety and post-intervention pain. Next, using the PROCESS macro for SPSS, we conducted a bias-corrected mediation analysis using 5,000 bootstrapped resamples to examine the role of post-intervention anxiety as a potential mediator of the group difference in post-intervention pain severity, controlling for patient characteristics that showed group differences at baseline. Estimates of indirect effects were considered significant when zero was not included in the 95% confidence intervals.

## RESULTS

### Patient Characteristics

**Figure 1:**
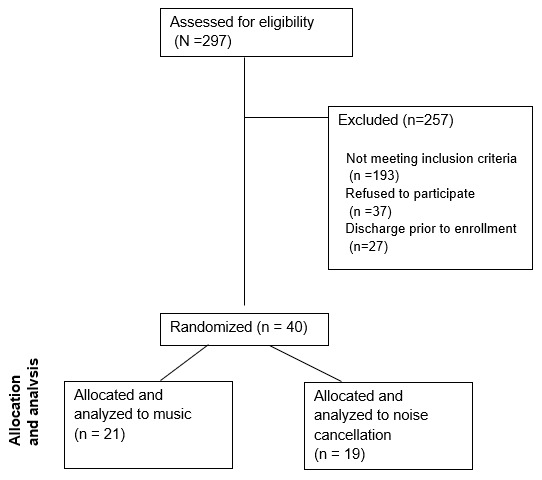
Study flow diagram for effect of music on musculoskeletal low back pain in the emergency department.

We screened 297 individuals, of whom 104 were eligible (Figure 1). We enrolled 40 participants between July–October 2022. Common reasons for nonparticipation were lack of interest in research or too much pain. There were 40 patients with an average age of 47.2 years (SD 16.9, range: 21–81) and 27 (68%) were female. Seventeen participants identified as White (42.5%), 11 as Black (27.5%,), two as Asian (5%), one Native Hawaiian or Pacific Islander (2.5%,), two more than one race (5%), four “other” (10%), three (7.5%) did not report their race, and of all participants, 10 (27%) identified as Hispanic.

A total of five (13%) patients self-reported using opioids, and 11 (28%) reported using non-opioid analgesics prior to their ED visit. Prior to the interventions, patients reported an average pain severity rating of 7.5/10 (SD 1.7) and an average anxiety rating of 5.7/10 (SD 3.1). While in the ED, 14 patients (35%) received opioids, and of these patients, they received 19.6 MMEs on average (SD 11.1, range 4–38). Additionally, 36 patients (90%) received some type of non-opioid analgesic in the ED, including oral acetaminophen or NSAID, or topical NSAID or lidocaine. Patients were in the ED for a mean of 6.4 hours (median 5.4, SD 3.3, range 1.6–15.8), and three patients (8%) were subsequently admitted to the hospital. At discharge, 11 patients (38%) received a new opioid prescription, two (5%) continued taking the opioid prescription they were previously prescribed, and 29 (73%) received a prescription for a non-opioid analgesic.

### Group Differences in Baseline Characteristics

Twenty-one patients were randomized to the music group and 19 patients to the noise cancellation group. Patients randomized to the music group reported significantly higher baseline trait pain catastrophizing (PCS) compared to patients randomized to the noise cancellation group (mean ±SD: 28.4±12.6 vs 19.4±10.8, *P* = .02). There were no other significant differences between the two groups based on any other baseline psychosocial factors, demographic characteristics, or medication use prior to visiting the ED (Table 1). We also did not observe a significant difference in baseline pain severity or anxiety ratings between the music and noise cancellation groups (Table 1).

### Group Differences in Post-Intervention Pain and Anxiety

**Figure 2:**
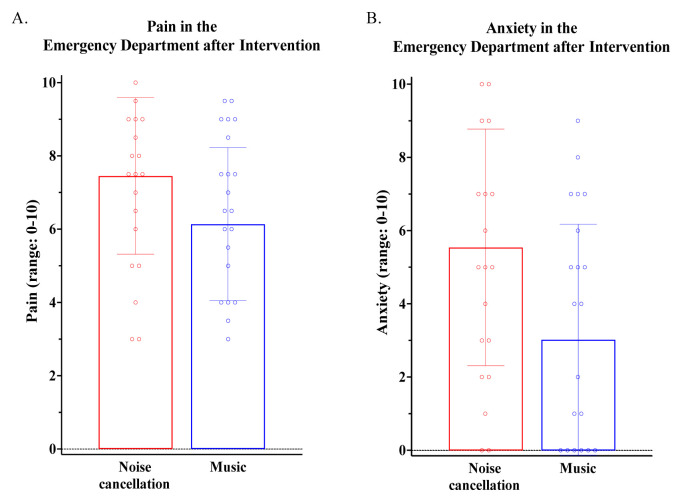
Differences in post-intervention (A) pain and (B) anxiety based on intervention group, controlling for baseline PCS. *PCS*, pain catastrophizing scores.

While in the ED, there were no significant group differences in the proportion of patients who were prescribed opioid or non-opioid analgesics, nor in the amount of opioids (MMEs) prescribed. We conducted two ANCOVAs to examine whether post-intervention pain severity and anxiety significantly differed between the music and noise cancellation groups, while controlling for baseline trait PCS. There was a significant main effect of intervention on pain severity [*F* (1,37)= 4.69, *P* = 0.037, η_p_^2=^.11], such that patients in the music group reported significantly lower pain severity scores compared to patients in the noise cancellation group (estimated mean ± SE: 6.1 ± 0.4 vs 7.5 ± 0.4, *P* = 0.037) (Figure 2A). Similarly, there was a significant main effect of intervention on anxiety [*F*(1,36) = 6.40, *P* = 0.016, η_p_^2=^.15], with patients in the music group reporting significantly lower anxiety scores than patients in the noise cancellation group (3.0 ± 0.7 vs. 5.5 ± 0.7, *P* = 0.016) (Figure 2B).

### Exploratory Analysis: Post-Intervention Anxiety as a Mediator of the Group Difference in Post-Intervention Pain Severity

**Figure 3:**
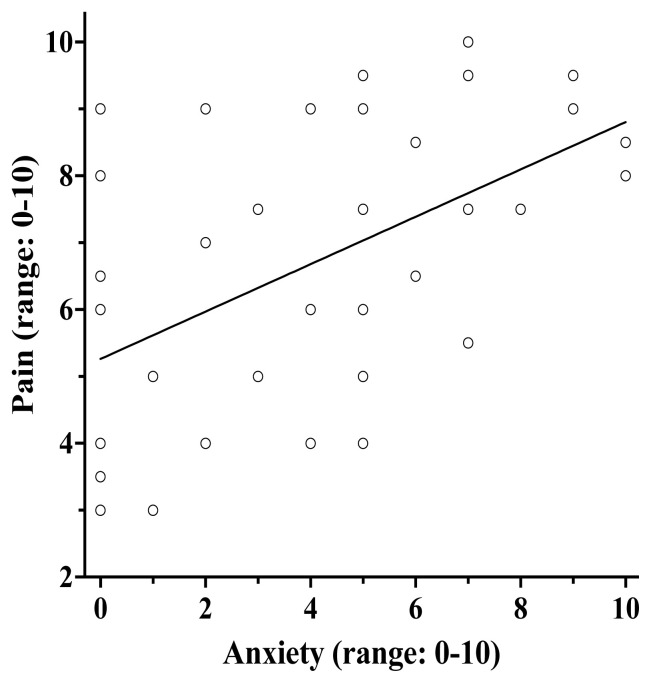
Greater post-intervention anxiety was correlated with greater post-intervention pain (*r*=0.54, *P* <0.001).

**Figure 4:**
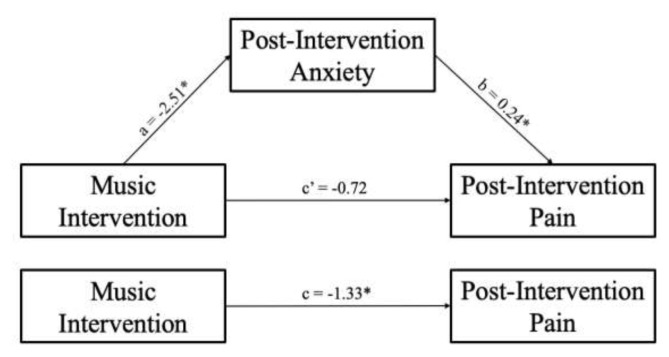
The mediating effect of post-intervention anxiety in the relationship between the music intervention and post-intervention pain, controlling for baseline Pain Catastrophizing Scale score * *P* <.05.

A correlation analysis showed that greater post-intervention anxiety was significantly associated with greater post-intervention pain severity (*r*=0.54, *P* <0.001) (Figure 3). Since we found that music modulated post-intervention anxiety, we were interested in exploring whether lower levels of anxiety post-intervention among patients in the music group could partially explain, or mediate, the group difference in pain severity post-intervention. A mediation analysis was conducted with intervention group entered as the independent variable (*x* variable), post-intervention anxiety as a mediator (*m* variable), and post-intervention pain severity as the outcome variable (*y* variable), controlling for baseline PCS (Figure 4). The overall model predicting post-intervention pain severity was significant *F*(3,35) = 8.12, *P* < .001, *R^2^* = 0.41. Importantly, there was a significant indirect effect of intervention group on post-intervention pain severity through post-intervention anxiety (*b* = −0.60, 95% CI [−1.33, −0.09]). The direct effect of intervention group (*b*=−1.33, *P*=.04, 95% CI [−2.59, −0.06]) on post-intervention pain severity was no longer significant when post-intervention anxiety was included in the model (*b* = −0.72, *P* = .26, 95% CI [−2.01, 0.56]). This suggests that patients who listened to music reported lower post-intervention anxiety, and, in turn, lower post-intervention pain severity.

### Post-Intervention Characteristics

We wanted to gain insight into patients’ experience with music both prior to participating in our study, as well as their impressions of use of music in the ED during our study. Most patients reported that they had used music or a music app for relaxation in the past (63%), with 38% of patients reporting that they typically spend 30–60 minutes listening to music every day. Patients were asked how much they liked the music/noise cancellation, and those who listened to music reported that they liked the music significantly more than those who used noise cancellation headphones (5.0 ± 0.2 vs. 3.7 ± 1.3, *P* <.001). Of the 21 patients in the music group, 40% reported that they perceived improvement in their pain after the intervention and 60% reported no perceived change. Of the 19 in the noise cancellation group, 16% reported that they perceived improvement in their pain after the intervention, 74% reported no perceived change in their pain, and 10% reported that their pain worsened. Notably, there were no differences in the amount of opioid medications administered in the ED or prescribed at discharge between the groups (*P*-values>.05). The majority of patients also reported that they thought they could deal with their pain if they had music or noise cancellation in combination with taking non-opioid medications (54%) (ie, using music as an adjuvant analgesic).

## DISCUSSION

Few non-pharmacologic analgesic options exist that can be easily, inexpensively, and flexibly deployed in the ED. Music, given its unique trans-cultural applicability and potential targets in the biopsychosocial model of pain, is an enticing adjunctive intervention to address the experience of pain in the ED. The present investigation demonstrated that patients who listened to self-selected music experienced less pain and anxiety post-intervention than patients who used noise cancellation headphones alone. Furthermore, we found that patients who listened to self-selected music reported lower post-intervention anxiety, which partially contributed to why they reported lower post-intervention pain severity. These findings suggest that music may be a helpful adjunctive therapy in the ED to address pain by decreasing emotional distress.

Previous experimental studies demonstrated the effectiveness of listening to music to address acute painful conditions.^12^,^13^,^19^,^20^ Within the clinical context, one randomized controlled trial (RCT) involving patients in the ED with simple lacerations demonstrated that listening to participant selected music from a pre-curated panel significantly reduced pain and anxiety associated with the procedure and resulted in an improved ED experience. 19,20 In another randomized study, listening to ambient music significantly decreased pain and anxiety in individuals with diverse chief complaints related to pain.^21^ Similarly, our previous pilot study demonstrated that prescribed, brief sessions of a relaxing music app can decrease anxiety with regard to pain in the ED.^12^ Taken together, these studies indicate that various types of music show efficacy in both experimental and clinical settings, including among patients in the ED, and are likely feasible to employ.

As all the clinical studies also included other analgesic use as per clinical practice, it seems likely that music may be best used as an adjunctive intervention to assist in managing pain and anxiety while in the ED. Similarly, the majority of our patients received multimodal analgesia including acetaminophen, NSAIDs, and lidocaine patches. In post-intervention surveys, about half of our participants (54%) endorsed that they thought they could manage their pain if they listened to music in combination with taking non-opioid medications, suggesting that music is an acceptable adjunctive strategy to help patients cope with their pain. Although we found no difference between the groups in opioid medication administration in the ED or prescribed at discharge, it is reasonable to consider using music as an adjunct for pain. Based on this data, clinicians may consider adding a brief music session in individuals with acute back pain, either as a protocol or on an individualized basis, among patients that demonstrate or report higher anxiety.

Our main analysis suggested a relatively significant impact of listening to music on anxiety itself (greater overall reduction than the reduction in pain scores), consistent with the idea that one mechanism of music’s analgesic impact could be through anxiety reduction. Anxiety is a known positive modulator of pain severity in both acute and chronic pain settings.^22^,^23^ Our exploratory analysis demonstrated that lower levels of post-intervention anxiety partially contributed to why patients who listened to their self-selected music reported lower post-intervention pain severity compared to those who used noise cancellation. From a clinical perspective, this may suggest that individuals who report higher degrees of anxiety surrounding back pain could derive greater benefit from music therapy. Reducing patients’ anxiety may be especially beneficial for improving acute pain symptoms.

The acute environment of the ED, uncertainty about the initial diagnosis of musculoskeletal back pain, and fear surrounding disability and mobility likely contribute to increased anxiety surrounding pain in the ED compared to other clinical settings. Addressing these factors using a music intervention may indirectly impact the experience of pain, and thereby serve as an adjunctive strategy when pharmacologic options are exhausted or used in advance of certain maneuvers such as ultrasound-guided nerve blocks. Interestingly, despite the significant effect of music on anxiety and pain that we observed in this study, we did not observe group differences in the amount of opioid analgesics prescribed while in the ED or at discharge. This lack of effect on opioid administration may be due to changes in our practice surrounding opioid prescribing for musculoskeletal back pain at this academic site. Specifically, we have dramatically reduced the overall use and dose of opioids employed, compared to historical practices, which is likely why a similar proportion of patients in each group received opioid and non-opioid analgesics to manage their pain.

Given the promise of music as an adjunctive therapy for musculoskeletal back pain the ED, there are several future directions that should be investigated. The selection of type and method of music delivery remains heterogenous across studies. It is likely that each strategy of music (eg, researcher selected vs participant selected), type (vocals vs instrumental vs other sounds) and delivery method (participant generated vs listening to music) may result in different effects. Some of these modalities, like user-generated or group-based music therapy, are infeasible in the ED setting, but other strategies such as a pre-curated “relaxing” playlist compared to asking patients to select their own music may be strategies that can be integrated into ED operations. Self-selection may have some benefits over a pre-curated playlist due to the subjective nature of music preference. As people tend to have a good sense of the type of music they enjoy, it is unlikely that a patient would deliberately administer an ill-fitting kind of music.

It is possible that providing access to a streaming music service for patients in the ED may help address some acutely painful events, but given the significant role of music in modulating anxiety, it may also assist with the overall milieu of the ED. Additionally, there may be indirect benefits of providing music interventions in the ED related to patient experience and satisfaction. Further research that investigates both the type and delivery method of music interventions, the patients they most benefit, and their impact on ED experience are warranted. Perhaps most appealing, if future studies demonstrate efficacy of music interventions on certain painful experiences in the ED, the threshold to implement these options may be lower compared to other interventions. For example, an ED could stock headphones and a streaming music device that could be cleaned between use, or clinicians could guide patients to access guided music interventions on their own phones.

## LIMITATIONS

This investigation had several limitations. First, we conducted our study at a single, tertiary, urban, academic teaching hospital where the majority of patients were evaluated by residents or physician assistants in conjunction with attending physicians. Practices and expectations surrounding analgesia for musculoskeletal back pain may vary across different sites with different practice models. Additionally, we did not collect information on the location of patients within the ED (eg, waiting room, private room, etc), which should be considered in future studies. Second, we opted to allow participants to select their own choice of music in this study. The degree to which these findings may generalize to use of standardized music interventions, chosen by the research team, is unknown. Similarly, the relative efficacy of different specific types of music (eg, instrumental or vocals, different genres of music) among individuals with pain in the ED was not specifically tested. It seems likely, based on previous studies,^24^ and, based on the fact that our subjects did choose a variety of different music, that musical tastes vary, and allowing patient choice of music may be both the most pragmatic and efficacious route of music administration. Additionally, the intervention was brief, which may have limited the effect seen. Finally, we selected a relatively common condition where patients are likely to be discharged and the expected course of illness is relatively brief. The effect of music interventions on other painful conditions may, therefore, vary.

## CONCLUSION

A brief, patient-selected music intervention resulted in significantly less pain and anxiety compared to the use of noise cancellation among individuals presenting to the ED with musculoskeletal back pain. Additionally, participants who listened to their self-selected music reported lower levels of anxiety post-intervention, which in turn, contributed to lower levels of pain. This data suggests that listening to music in the ED may be a low-threshold adjunctive tool in the armamentarium of non-pharmacologic interventions for musculoskeletal back pain.

## Supplementary Information







## Figures and Tables

**Table 1:** Patient characteristics.

	Music group (n=21)	Noise cancellation group (n=19)	P-value
	M±SD or n(%)	M±SD or n(%)	
Demographics			
Age	44.3±16.1	50.3±17.7	.27
Sex			.34
Male	5 (24%)	7 (37%)	
Female	16 (76%)	11 (58%)	
Non-binary/non-conforming	-	1 (5%)	
Race			.60
White	8 (38%)	9 (47%)	
Black	8 (38%)	3 (16%)	
Asian	1 (5%)	1 (5%)	
Native Hawaiian or Pacific Islander	-	1 (5%)	
More than one race	1 (5%)	1 (5%)	
Other	1 (5%)	3 (16%)	
Unknown/not reported	2 (10%)	1 (5%)	
Diagnosis of depression	1 (5%)	-	.34
Diagnosis of anxiety	1 (5%)	1 (5%)	.97
Baseline pain and psychosocial factors			
BPI pain intensity (range:0–10)	6.9±1.6	7.3±1.9	.41
BPI pain interference (range:0–100)	49.6±16.3	48.5±16.8	.84
PROMIS anxiety (range:7–35)	20.1±8.5	17.1±7.08	.24
PROMIS depression (range:7–35)	17.5±7.5	14.9±6.3	.24
PROMIS sleep (range:8–40)	27.8±8.0	28.0±5.4	.93
Perceived stress (PSS) (range:0–40)	18.1±8.4	16.3±6.6	.48
Trait pain catastrophizing (PCS) (range:0–52)	28.4±12.6	19.4±10.8	.02
Pre-intervention			
Pain rating	7.4±1.7	7.6±1.9	.73
Anxiety rating	5.2±3.4	6.3±2.7	.31
Medication use			
Prior to ED Visit			
Taking opioids	2 (9.5%)	3 (16%)	.55
Taking non-opioid analgesics	4 (19%)	7 (37%)	.21
While in the ED			
Prescribed opioids	9 (43%)	5 (26%)	.27
MMEs	18.5±11.2	21.6±11.9	.64
Prescribed non-opioid analgesics	20 (95%)	16 (84%)	.25
At discharge from ED			
Prescribed opioids	8 (38%)	5 (26%)	.44
MMEs	38.9±25.0	43.8±31.0	.76
Prescribed non-opioid analgesics	14 (67%)	15 (79%)	.39

*BPI*, Brief Pain Inventory; *PROMIS*, patient-reported outcome measurement information system; *ED*, emergency department; *MMEs*, morphine milligram equivalents.
