# Supplementary material for: Randomized Trial of Self-Selected Music Intervention on Pain and Anxiety in Emergency Department Patients with Musculoskeletal Back Pain
Source: West J Emerg Med. 2025 Jun 25;26(4):1112–9. doi: 10.5811/westjem.34871 (PMC12342572; doi:10.5811/westjem.34871)
Supplement: Supplementary file 1 [file wjem-26-1112-g002.pdf]

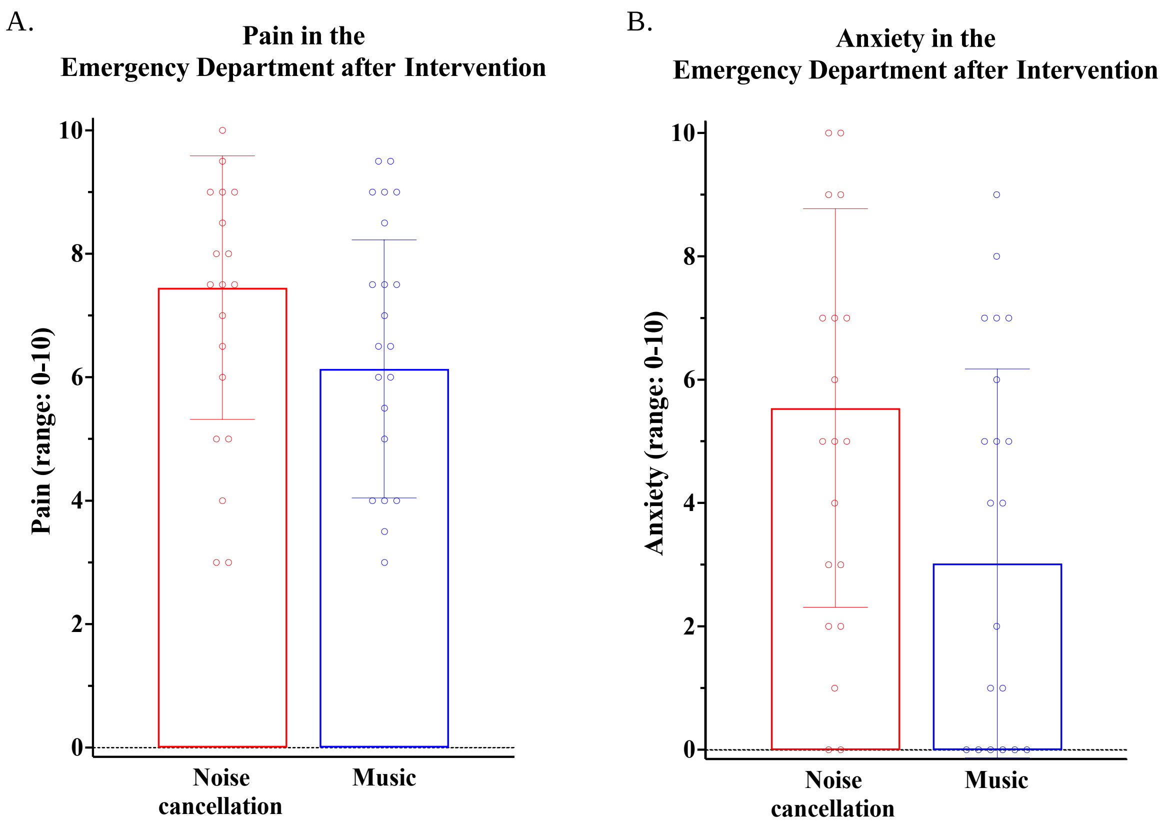
A.
Pain in the
Emergency Department after Intervention
Pain (range: 0-10)
10
8
6
4
2
0
Noise cancellation
Music
B.
Anxiety in the
Emergency Department after Intervention
Anxiety (range: 0-10)
10
8
6
4
2
0
Noise cancellation
Music
