# Supplementary figures and images for: Randomized Trial of Self-Selected Music Intervention on Pain and Anxiety in Emergency Department Patients with Musculoskeletal Back Pain
Source: West J Emerg Med. 2025 Jun 25;26(4):1112–9. doi: 10.5811/westjem.34871 (PMC12342572; doi:10.5811/westjem.34871)

## Correlation between Post-Intervention Anxiety and Pain

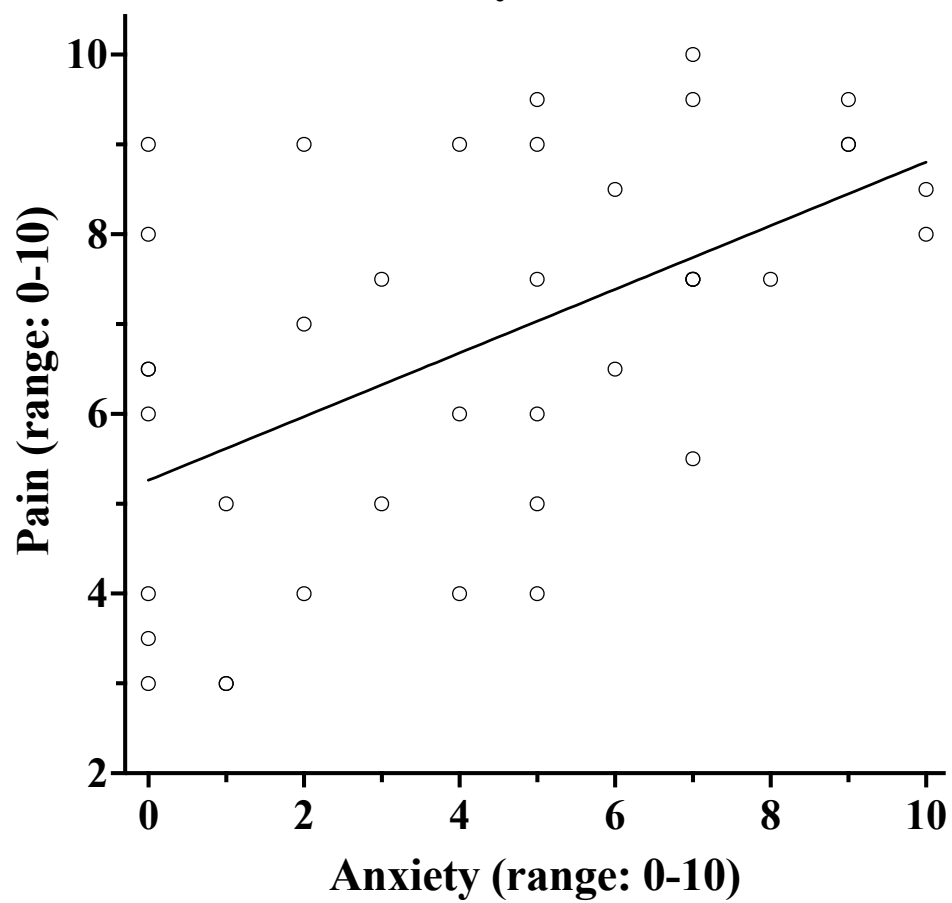

Supplement: Supplementary file 2 [file wjem-26-1112-g003.pdf]

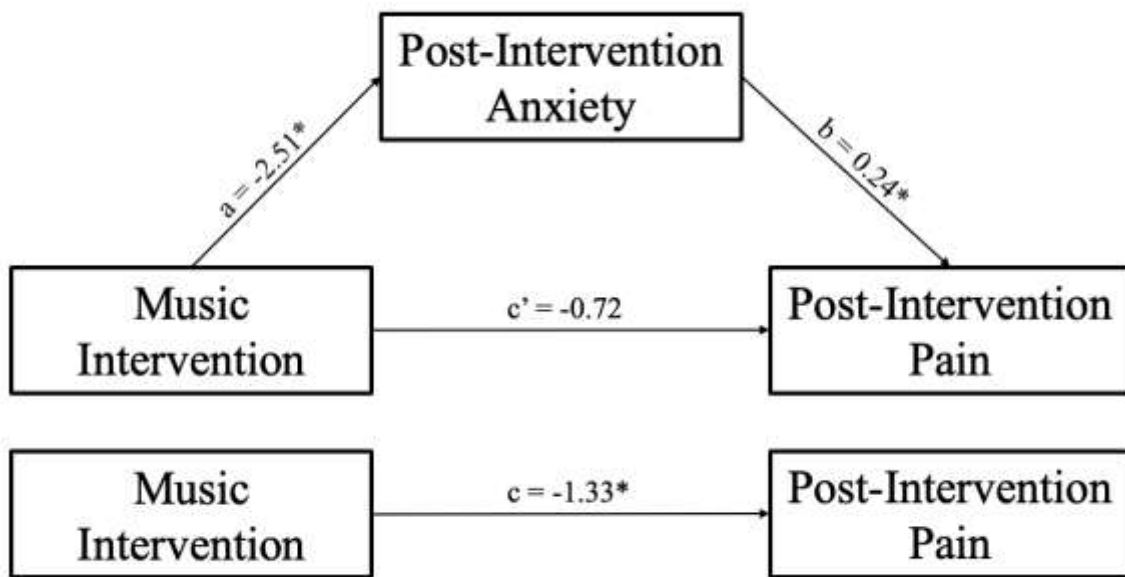

Supplement: Supplementary file 3 [file wjem-26-1112-g004.pdf]
